# Nutritional Assessment in Preterm Infants: A Practical Approach in the NICU

**DOI:** 10.3390/nu11091999

**Published:** 2019-08-23

**Authors:** Luis Pereira-da-Silva, Daniel Virella, Christoph Fusch

**Affiliations:** 1 Medicine of Woman, Childhood and Adolescence, Faculdade de Ciências Médicas|NOVA Medical School, Unversidade Nova de Lisboa, 1169-056 Lisbon, Portugal; 2 Dietetics and Nutrition, Lisbon Polytechnic Institute, Lisbon School of Health Technology, Av. Dom João II MB, 1990-094 Lisbon, Portugal; 3 NICU, Hospital Dona Estefânia, Centro Hospitalar Universitário de Lisboa Central, 1169-045 Lisbon, Portugal; 4 Nuremberg General Hospital, Department of Pediatrics, Paracelsus Medical University, Prof.-Ernst-Nathan-Straße 1, 90419 Nürnberg, Germany

**Keywords:** anthropometry, biochemical markers, body composition, growth charts, nutritional assessment, preterm infant

## Abstract

A practical approach for nutritional assessment in preterm infants under intensive care, based on anthropometric measurements and commonly used biochemical markers, is suggested. The choice of anthropometric charts depends on the purpose: Fenton 2013 charts to assess intrauterine growth, an online growth calculator to monitor intra-hospital weight gain, and Intergrowth-21st standards to monitor growth after discharge. Body weight, though largely used, does not inform on body compartment sizes. Mid-upper arm circumference estimates body adiposity and is easy to measure. Body length reflects skeletal growth and fat-free mass, provided it is accurately measured. Head circumference indicates brain growth. Skinfolds estimate reasonably body fat. Weight-to-length ratio, body mass index, and ponderal index can assess body proportionality at birth. These and other derived indices, such as the mid-upper arm circumference to head circumference ratio, could be proxies of body composition but need validation. Low blood urea nitrogen may indicate insufficient protein intake. Prealbumin and retinol binding protein are good markers of current protein status, but they may be affected by non-nutritional factors. The combination of a high serum alkaline phosphatase level and a low serum phosphate level is the best biochemical marker for the early detection of metabolic bone disease.

## 1. Introduction

Growth faltering has been extensively documented in infants born very prematurely, being attributed to inadequate nutrient intake and lack of standardization in feeding practices [1,2]. Poor postnatal growth is associated with adverse neurocognitive outcomes [3]. Although fast postnatal growth is associated to better neurodevelopment in preterm infants, early fast weight gain may be associated with later obesity, high blood pressure, and adverse cardiovascular and metabolic outcomes [3,4,5]. Both undernutrition and overnutrition may predispose to obesity and cardiovascular disease [6,7].

Monitoring nutritional status is required to detect nutritional deficits early and to guide nutrition support in preterm infants under intensive care. Thus, nutritional assessment should be an essential skill of neonatal staff caring for preterm infants [8].

Tracking body composition rather than just body weight is more accurate for monitoring nutritional status and improving nutritional outcomes [9,10]. Dual-energy X-ray absorptiometry (DXA) and air displacement plethysmography (ADP) are validated and convenient methods for body composition assessment in small infants, since they are noninvasive, rapid to perform, and not affected by movements [10,11,12]. However, these methods are expensive and not accessible for the majority of clinical settings [10,13,14]. As an alternative, anthropometry may be a proxy for body composition [14]. This is a noninvasive and inexpensive method, suitable for bedside evaluation, despite limitations in the validity of several measurements in small infants [15,16]. In addition, most anthropometric prediction equations were developed based on cross-sectional studies at and near birth [12].

A comprehensive approach for the evaluation of nutritional status includes anthropometry, biochemical markers, clinical parameters, and dietary assessment [8]. While clinical signs of early malnutrition are largely imperceptible, certain clinical biochemical markers can provide a useful insight into nutritional status, particularly when used as a complement to anthropometric measurements [8,17].

Tools for the nutritional assessment of preterm infants under intensive care are herein reviewed from a practical approach, focusing on the most commonly used anthropometric measurements and biochemical markers. Accuracy and reference values for each indicator are revisited. Clinical and dietary assessments, which are a part of nutritional assessment, are beyond the scope of this review.

## 2. Anthropometry

In preterm infants, anthropometry is useful for several purposes, including the diagnosis of fetal malnutrition and risk assessment of early metabolic complications, monitoring growth during hospital stay, and early recognition of undernutrition or overnutrition [16].

Revisited direct anthropometric measurements include body weight and length, head circumference (HC), mid-upper arm circumference (MUAC), and skinfolds [12,16]. Indices calculated from direct measurements have been proposed, assuming that some may provide a better insight into body composition than the original direct measurements [15,16]. Derived measurements herein revised include equations based on body weight and length, MUAC to HC ratio (MUAC:HC), and mid-arm cross-sectional areas.

Accurate anthropometry should rely on appropriate technique and instrumentation, and the obtained results interpreted by plotting on appropriate growth charts or comparing with appropriate reference values [11,16].

### 2.1. Body Weight

Body weight measurement is simple and reproducible, and it is the most used single parameter to monitor neonatal growth and nutritional status in clinical practice [18]. However, body weight does not give any information on the size of body compartments and, consequently, on the quality of growth [16,19]. In some preterm infants, weight gain may result from a positive balance of water and sodium rather than the accretion of fat or protein, as happens in sick infants affected by edema [18,20]. Nevertheless, in preterm infants measured shortly after birth, body weight unadjusted for length was found to be strongly associated (*r*^2^ = 0.97) with ADP-determined fat-free mass [13]. It is not surprising that body weight is a good proxy of lean mass particularly in very- and extremely preterm infants since they are born with scarce fat mass [13,21]. In the fetus, body fat changes from a minimum deposit during the first trimesters of gestation to an exponential increase after 30 weeks, period in which 94% of all fat deposition occurs [22].

#### 2.1.1. Classification by Birth Weight

Regardless of the gestational age and based only on birth weight, neonates overall are classified as macrosomic if they are born >4000 g, low birth weight if born <2500 g, very low birth weight if born <1500 g, and extremely low birth weight if born <1000 g. This classification bares the risk of neglecting underlying physiology; for instance, an infant born with 31 weeks of gestation and 1500 g, normally grown but immature, may have different clinical problems from a mature infant with 37 weeks of gestation but severely malnourished. To assess nutritional status at birth, a classification relating birth weight with gestational age is more appropriate; accordingly, neonates are classified as large- (LGA), appropriate- (AGA), and small-for-gestational age (SGA) [16]. There is no consensus regarding the cut-offs for this classification. While some authors define the 10th and 90th percentiles as lower and higher thresholds, others consider as lower thresholds the 5th percentile, 3rd percentile, or -2 standard deviations to classify as SGA, and the 95th percentile, 97th percentile or +2 standard deviations as higher thresholds to classify as LGA [23]. Due to the skewed distribution of weight by gestational age, the use of reference curves based on percentiles is preferred to those based on average and standard deviations [24]. The ability to estimate statistically defined thresholds is dependent on the sample size for each gestational group of interest; only samples greater than 120 individuals have enough statistical power to define the 3rd or the 5th percentiles [25,26,27]. The definition of clinically significant thresholds requires prospective studies rather than cross-sectional assessments [28]. There is evidence that, for infants born at term, using any of the usual criteria, being small or large size for gestational age at birth is not sensitive or specific enough to identify infants at risk of adverse neonatal outcomes related with abnormal intrauterine growth [29]. There are SGA infants that are not growth-restricted and AGA infants that suffered intrauterine growth restriction [11,30,31,32]. Such evidence is lacking for prematurely born infants. Intrauterine growth restriction is defined as a pathologic process that causes birth weight to be less than that genetically expected, although this potential is difficult to assess [32,33]. Similarly, there is no universal consensus on the LGA classification, in part due to the secular trend in the proportion of women giving birth to large infants, attributed to increases in maternal height, body mass index, gestational weight gain, and diabetes [28,34].

#### 2.1.2. Technique and Instrumentation

Infants should be weighed naked, ideally on an electronic scale calibrated to the nearest gram. In the neonatal intensive care unit, the accuracy is improved if the infant is measured disconnected from the ventilator and immediately prior to a feed. Inaccuracies usually result from drips and inserted tubes touching the mattress, the presence of a full bladder, and infants’ agitation [12,16].

To minimize handling of fragile or sick preterm infants, modern incubators are fitted with digital weighing scales [12]. Excellent precision and reproducibility were described for digital electronic weighing scales compared with beam scales, reducing the observer bias and reading errors [35].

#### 2.1.3. Reference Values

Appropriate charts and standards relating body weight with the infant’s age should be used depending on the purpose [15,36] (Table 1).

##### To Assess Intrauterine Growth Based on Birth Weight

The cross-sectional sex and gestational age specific Fenton 2013 charts [37] are appropriate. These are based on a meta-analysis of six large population-based surveys of size at preterm birth, representing almost 4 million births from 22 to 36 weeks gestation. These cross-sectional preterm charts were harmonized with the longitudinal WHO Growth Standards for infants born at term [38], smoothing the data between the preterm and WHO estimates while maintaining integrity with the data from 22 to 36 weeks and at 50 weeks [37]. The portion of the curves between 37 and 50 weeks were validated by comparing them using weight gain patterns of contemporary preterm infants [39]. The Peditools.org chart (http://www.ucalgary.ca/fenton/ 2013chart) is an online clinical tool allowing the calculation of z-scores for the Fenton 2013 charts [37]. 

##### To Monitor Intra-Hospital Growth

Charts that do not take into account the initial physiological weight loss, such as the Fenton 2013 charts, are not appropriate to monitor short-term postnatal growth [40,41]. A recent large, international longitudinal observational study showed that preterm infants with uncomplicated postnatal adaptation have transitioned to a weight gain trajectory of 0.8 standard deviations below birth weight at the 21th postnatal day [41]. Based on these data [41,42], a free-access online growth calculator (https://www.growthcalculator.org/) was developed displaying graphically in which percentile the current weight is plotted, also providing the target weight and deviation of the current weight in grams.

Weight gain velocity (in g/kg/day) over the previous days is more sensitive in identifying changes in growth than examining the weight plotted on growth curves [18]. The period allowing a precise calculation of weight gain velocity seems to be a time interval of at least 5–7 more days [43]. A recent analysis of growth velocity curves concluded that body weight rates of 15–20 g/kg/d are a reasonable goal for infants born at 23–36 weeks gestation [44]. An exponential model to calculate weight gain velocity was proposed as a more accurate estimate, not affected by decreasing birth weight and increasing length, compared with the conventional two-point assessment method [45]. Furthermore, the development of exponential models indicates that the individualized monitoring of weight gain velocity may be more accurate than plotting on reference curves [24].

##### After Discharge

For infants born at more than 26 weeks and less than 37 weeks gestation, the longitudinal Intergrowth-21st standards [46] were designed to follow up to 64 weeks postmenstrual age. For the development of these charts, a cohort of 4607 healthy pregnant women from eight geographical areas of the world were enrolled, and fetal growth was monitored from 14 weeks gestational age to birth [47]. These charts only included 201 preterm infants who met the criteria to be considered healthy and stable have been followed up [46], and the small sample size is a limitation.

### 2.2. Crown-Heel Length

Body length reflects skeletal growth and fat-free mass [32,35]. In preterm infants, it was found to be a good predictor (*r*^2^ = 0.85) of ADP-determined fat-free mass shortly after birth [13].

Length assessment is useful only if measurement has been accurately undertaken [32]. Length is frequently measured inaccurately or ignored in clinical practice because of the perceived difficulty in measurement of neonates [35,48]. The reluctance of the observer to extend the lower limbs forcefully is a factor affecting its accuracy in full-term neonates, who are more comfortable in flexion [48,49,50,51]. In spite of the lack of similar studies in preterm infants, the reluctance to fully extend the lower limbs as recommended seems to be widespread in these infants. Moreover, the accuracy of measured length is of utmost importance when it is squared or cubed in indices, since a small error in its measurement amplifies the distortion of final results [16,52]. 

#### 2.2.1. Technique and Instrumentation

Recumbent crown-heel length should be measured weekly to the nearest millimeter with the infant in supine position, by two collaborators, preferably involving a parent [16,53]. One collaborator holds the infant’s head against the fixed headboard, aligned with the trunk; the other extends completely the lower limbs by gently pressing the infant’s knees down, holds the feet vertically at a right angle to the length board, and moves the footboard up against the heels [16]. An alternative validated method, with similar accuracy and causing significantly less discomfort, consists of measuring length with only one lower limb extended, provided trunk and limb axes are parallel and the line between the ileac crests is at a right angle with the trunk axis [16].

Tape measurements are of doubtful reliability [19,48]. A prematurometer was designed as the more appropriate device to measure preterm infants’ length inside incubators [33], but proper application may be cumbersome [8]. As an alternative, simpler and less expensive rigid recumbent length boards providing accurate measurements are used in clinical practice [8,53].

#### 2.2.2. Reference Values

To assess length at birth, the cross-sectional sex and gestational age specific Fenton 2013 charts [37], which included 151,527 neonates with measured length, are appropriate. These charts can be used to monitor the linear growth of preterm infants from birth to 50 weeks postmenstrual age [16]. Body length velocity provides a more sensitive measurement of linear growth and a rate of approximately 0.9–1.1 cm/week is a reasonable goal, particularly for 23–30 weeks postmenstrual age [19,43].

After discharge, the longitudinal Intergrowth-21st standards [46] were designed to monitor linear growth up to 64 weeks postmenstrual age, for infants born at more than 26 weeks and less than 37 weeks gestation.

### 2.3. Head Circumference

An increase in occipitofrontal circumference reflects brain growth, although it is not a sensitive or specific measure [19]. Some factors should be taken into account when interpreting HC measurements in neonates. During the first postnatal week, HC may decrease by about 0.5 cm due to extracellular fluid space contraction [19]. If HC values are above or below the reference limits it may represent a variant of normal, and therefore, the parents’ HC should be measured and plotted [16].

In infants born prematurely, a non-expected deviation of head growth in the presence of good weight and linear growth should be investigated for causes other than inadequate nutrient intake. These may be related with prematurity-related morbidity, including post-hemorrhagic hydrocephalus or brain atrophy [19,31]. In addition, magnetic resonance imaging measurements recently revealed that in “encephalopathy of prematurity”, increased size of the extra-axial spaces may artificially increase HC [54].

In preterm infants, postdischarge head growth seems to be a better predictor of cognitive outcomes than intra-hospital head growth [55].

#### 2.3.1. Technique and Instrumentation

Head circumference should be measured weekly to the nearest millimeter, encircling the supraorbital ridges and occipital protuberance with a non-stretchable measuring tape. The largest occipitofrontal of three consecutive measurements should be considered [16,18]. In the presence of head molding or scalp edema at birth, measuring HC at 48–72 postnatal hours is suggested, allowing the reduction or resolution of the edema [16,35].

#### 2.3.2. Reference Values

To interpret HC measurements at birth, the cross-sectional sex and gestational age specific Fenton 2013 charts [37], which included 173,612 neonates with measured HC, are appropriate [37]. The same charts can be used to monitor the head growth of preterm infants up to 50 weeks postmenstrual age [16]. Head circumference growth velocity provides a more sensitive assessment of head growth and rates of 0.9–1.0 cm/week are a reasonable goal for preterm infants, particularly for 23–30 weeks postmenstrual ages [19,37].

After discharge, the longitudinal Intergrowth-21st standards [46] were designed to monitor head growth up to 64 weeks postmenstrual age, for infants born at more than 26 weeks and less than 37 weeks gestation.

### 2.4. Mid-Upper Arm Circumference

The MUAC reflects the combined arm muscle and fat; a decrease in its value indicates a reduction in body muscle and/or fat mass [32,56]. As the upper arm is less affected by fluid status changes than other areas of the body, in the presence of edema, the MUAC may be a more accurate estimate than other measurements [32].

In preterm infants, measurements of MUAC are reproducible and detect changes over time [56]. In moderately preterm infants, adiposity defined by ADP-determined percent of fat mass (%FM), accounts for 60.4% of the variation of MUAC [57].

#### 2.4.1. Technique and Instrumentation 

A flexible non-stretchable measuring tape with a width of 1.0 cm is appropriate for MUAC measurement in preterm infants [16,58].

Measurements are taken with the arm extended, at mid distance between the tip of the acromion and the olecraneon with the infant in dorsal decubitus and the arm lying laterally to the trunk [16,58]. In extremely premature infants, accurate MUAC measurement may be difficult due to inadvertent compression of the skin and underlying loose tissues when attempting to adjust the tape perfectly around the arm, or by small spaces left between the wrinkled skins when avoiding compression [59].

#### 2.4.2. Reference Values

The longitudinal grids published by Ehrenkranz et al. [60] are useful reference data, although they are not recent. These are based on a multicenter cohort of 1660 very low birth weight infants; the grids provide the average MUAC for each postnatal week, stratified by 100 g birth weight intervals.

### 2.5. Skinfolds

Skinfolds measurement is an inexpensive and suitable method for bedside nutritional assessment in neonates [10,16]. The triceps and biceps skinfolds are used mainly to assess peripheral subcutaneous fat, whereas subscapular and suprailiac skinfolds are used to assess central subcutaneous fat [61].

The use of skinfolds to estimate body fat assumes that the thickness of subcutaneous fat reflects a constant proportion of total body fat and the sites used for the measurements reflect the average thickness of the subcutaneous fat layer. Although these assumptions have been questioned [62], a good correlation was reported between skinfolds and DXA- [63,64] and ADP-determined body fat [57]. Contrarily, skinfolds seem to overestimate total body fat comparing with body water dilution measurements [65]; however, this method assumes a constant of lean tissue hydration that in fact is not constant and varies with age [10]. Some reasons have been reported for the discrepant results between studies. Skinfolds do not reflect intra-abdominal fat; the hydration status affects skinfold compressibility; and reproducible measurements require skill and practice of the observer [15,16,62,66]. Particularly in preterm infants, the rapidly changing distribution of fat accretion makes it difficult to generate an accurate equation for predicting total body fat [62].

#### 2.5.1. Technique and Instrumentation 

Appropriate calipers exerting a constant pressure of 10 g/mm^2^ of contact surface area should be used. Commonly used calipers are the Holtain (Holtain Ltd., Croswell, Crymych, UK) and the Harpenden (CMS Instruments, London, UK), with precisions of 0.1 and 0.2 mm, respectively [12,16]. The triceps skinfold is measured on the posterior surface of the extended arm, at the mid distance between the acromion and olecraneon. The biceps skinfold is measured on the anterior surface of the arm, at the same level as the triceps skinfold. The subscapular skinfold is measured at the lower angle of the scapula and the suprailiac skinfold immediately above the iliac crest, along the axis of the anterior mammillary line [16,63]. For measurement, the skinfold is pinched while pulling the fold away from the underlying muscle. The caliper is applied at a right angle to the raised fold, and the reading is taken 60 s after the application of the caliper [12,16]. Skinfolds should be collected in duplicate and a third measure is taken with the two closest measures averaged [12]. The practicality of using skinfold calipers in extremely preterm infants may be limited due to their size and underdeveloped skin [10].

#### 2.5.2. Reference Values

Sex and gestational age-specific references for triceps, biceps, subscapular, and suprailiac skinfolds were described for neonates born at 32 to 41 weeks gestation, measured shortly after birth [61,67]. There is insufficient information on longitudinal reference data of skinfolds for infants born preterm [68]; furthermore, it is yet unclear as to whether it is appropriate to use estimates of intrauterine growth reference for extrauterine growth [35].

### 2.6. Weight-to-Length Based Equations

Indices based on weight and length commonly used to assess body proportionality and body composition in neonates include the weight-for-length ratio, the body mass index (BMI) (weight/length^2^), and the ponderal index (weight/length^3^) [12,13,16].

The reliability of these indices is highly dependent on the accuracy of length measurement [16]. It has been described that accurate crown-heel length measurement is difficult to obtain at least in term neonates [51]. Inaccurate length squared in the BMI magnifies the error while leading to the index decreasing its ability to differentiate over- from underestimation. When cubed to obtain ponderal index, the inaccuracy of the length measurement is further magnified, despite the fact that it still differentiates overestimation from underestimation [52].

Ponderal index has been the traditional measure used to assess proportionality at birth and to distinguish between asymmetrical and symmetrical types of intrauterine growth restriction [69]. More recently, BMI has been reported to be more appropriate to assess body proportionality than either weight-for length ratio or ponderal index [70].

When used to assess body composition, BMI accounts for more than 81% of the variance of DXA-determined lean mass in AGA term infants measured shortly after birth [64]. Compared with ADP-determined %FM (adiposity), all weight-to-length based equations were found to be poor predictors of adiposity in preterm infants [10,13], even though the BMI z-score predicts adiposity better than the ponderal index (*r*^2^ = 0.43 vs. 0.29) [71].

#### Reference Values

Sex-specific reference curves for BMI at birth, obtained from 391,681 infants born at 22 to 42 weeks gestational age, have been published [72]. As tapes were used to measure length in this multicenter cross-sectional study, its accuracy for derived weight-to-length ratios have been questioned [52].

### 2.7. Mid-Upper Arm Circumference to Head Circumference Ratio

The MUAC:HC ratio may contribute to the estimation of body composition; its usefulness as a complementary index was assessed, comparing with DXA measurements [64]. This index was proposed to identify acute intrauterine malnutrition at birth, assuming that in acute protein-energy deprivation the brain is spared in relation to muscle and fat [73]. It may be particularly useful to identify symptomatic malnourished AGA neonates who are not diagnosed based exclusively on birth weight [74]. Longitudinal MUAC:HC measurements were reported to be useful for monitoring growth, seeming to not overestimate malnourishment in apparent protein-energy sufficiency [75].

#### Reference Values

Reference curves for MUAC to head circumference ratio were published for infants born at 25 to 42 weeks gestational age, measured shortly after birth [73]. These values have not yet been compared with a reference body composition assessment method, and no longitudinal reference values have been described in preterm infants.

### 2.8. Upper-Arm Cross-Sectional Areas

Upper-arm fat and muscle areas have been used in the assessment of nutritional status in infants [76]. For their calculation, it is assumed that the upper arm is cylindrical, the subcutaneous fat is a concentric ring evenly distributed around a circular core of muscle, the fat thickness is half the triceps skinfold, and the muscle includes the humeral diameter [77]. Two equations derived from MUAC and triceps skinfold have been proposed relying on different geometrical assumptions [77,78].

Upper-arm fat and muscle areas measurements should be interpreted with caution in neonates, since their ability to predict total body fat and muscle has been questioned [64]. In term infants assessed shortly after birth, the added value of cross-sectional arm areas contributed little to detect the variation of DXA-determined body lean and fat mass, compared with weight and length alone [64]. The ability of cross-sectional arm areas to predict mid-upper arm muscle and fat was also questioned. In full-term infants, a poor correlation of arm areas with ultrasound measurements was found, leading to overestimation of muscle and underestimation of fat [79]. In preterm infants, arm muscle and fat areas were inaccurate predictors (*r*^2^ < 0.56) of magnetic resonance imaging measurements [59]. These poor correlations may be explained by the limited reliability of MUAC [57] and triceps skinfold [65] measurements and by the oversimplification of geometrical assumptions used for the calculation of cross-sectional arm areas [16].

#### Reference Values

Longitudinal reference values for upper-arm cross-sectional areas have been reported only for infants born at term [80], although they have not been validated.

Advantages and limitations of the reviewed anthropometric measurements used for the assessment of nutritional status in preterm infants are summarized in Table 2.

## 3. Biochemical Markers

In preterm infants, some biochemical markers are useful in the assessment of nutritional status, helping to detect nutritional deficiencies before the appearance of clinical signs [17]. These markers should be interpreted with caution and used to complement other nutritional data, including anthropometric measurements [17].

Markers for metabolic and electrolyte, iron, protein, and bone status have been reviewed previously [8,17,81]. Markers to monitor protein status and bone status are summarized in Table 3.

### 3.1. Metabolic and Electrolyte Status

The detection of early postnatal metabolic complications requires monitoring of acid/base balance, glucose, electrolytes, calcium, phosphorus, and magnesium [8,17,81].

Preterm infants may have inadequate renal tubular reabsorption of sodium, and insufficient sodium supplementation is an underappreciated cause of poor growth [82]. Although serum sodium is the most commonly used measure of sodium status, urinary sodium better reflects total body sodium status. A urine spot with Na < 20 mEq/L associated with hyponatremia (<135 mEq/L), or a fractional excretion of sodium (FENa) < 4% are good indicators of body sodium depletion [83].

In stable growing infants receiving enteral nutrition, laboratory monitoring every week or every other week is sufficient [17]. In those receiving prolonged parenteral nutrition, liver function should be monitored using serum levels of direct bilirubin, aspartate amino transferase, alanine amino transferase, alkaline phosphatase, gamma-glutamyl transpeptidase, and triglycerides [17,84,85].

### 3.2. Iron Status

Cord ferritin lower than 75 ng/mL is associated with poor performance in motor skills and language development [86]. In stable growing preterm infants, inadequate iron supplementation necessary to support erythropoiesis results in suboptimal iron status, although multiple non-nutritional reasons contribute to iron deficiency [87,88]. Serum ferritin is commonly used to customize iron supplementation in growing preterm infants; however, being an acute phase protein, it may be inaccurate as an isolated biomarker [89]. Other iron status parameters frequently used in clinical practice include hemoglobin, serum iron, and total iron-binding capacity [89].

Reticulocyte hemoglobin content is a novel pre-anemia biomarker of iron deficiency described in healthy infants born at term [90] and its relevancy in preterm infants need to be explored.

### 3.3. Protein Status

Common markers used to assess protein status include blood urea nitrogen (BUN), serum prealbumin and retinol-binding protein (RBP) [8,17,18] (Table 3).

Serum albumin has a half-life of around 20 days; thus, it should not be used to monitor current protein status [8]. Likewise, serum total protein level is not a suitable indicator of protein status, since the variability of many proteins, particularly acute phase proteins, are independent of nutritional status [8].

#### 3.3.1. Blood Urea Nitrogen

In the early postnatal period, high BUN levels can result from factors other than protein intolerance, such as renal function, hydration status, and catabolism [17,91].

In clinically stable preterm infants with adequate hydration and normal renal function, BUN may be useful to monitor the adequacy of protein intake during enteral nutrition [92]. A BUN value lower than 1.6 mmol/L (4.48 mg/dL) indicates insufficient protein intake [8]. Specifically, BUN may guide the use of human milk fortifiers, and the addition of modular protein in the presence of poor growth [17,92]. An elevated BUN is more difficult to interpret; it may represent either amino acid intolerance or in presence of appropriate amino acid intake increased amino acid oxidation potentially as a consequence of insufficient energy intake as reported for breast milk (e.g., with low fat content) [93,94,95].

#### 3.3.2. Serum Prealbumin (Transthyretin)

Prealbumins are transporter proteins. Considering their short half-life of approximately 2 days, low levels may reflect current protein deficit [17,18,96]. In the presence of inflammation or infection, prealbumin levels may decrease; on the other hand, its synthesis is increased by exogenous steroids [97]. In these conditions, it may not be a reliable marker of protein status [97].

The reference values [mean (SD)] for prealbumin in preterm infants are 7.0 (1.7) mg/dL at birth, 9.5 (3.3) mg/dL at the 14th postnatal day, and 8.7 (2.3) mg/dL at the 28th postnatal day [98].

#### 3.3.3. Serum Retinol Binding Protein

Retinol-binding protein transports vitamin A. Considering its half-life of approximately 12 h, low levels may reflect current protein deficit [18,96]. In preterm infants, RBP levels should be interpreted with caution since they may be influenced by suboptimal iron, zinc, or vitamin A status [18,81]. RBP provides equivalent information to prealbumin, but its assay is more expensive [99].

The reference values [mean (SD)] for RBP in preterm infants are 1.3 (0.4) mg/dL at birth, 1.9 (1.0) mg/dL at the 14th postnatal day, and 1.6 (0.8) mg/dL at the 28th postnatal day [98].

#### 3.3.4. Serum Transferrin

Serum transferrin binds and transports iron in plasma. It should only be used as marker of protein status if body iron content is appropriate. In iron deficiency, transferrin concentration increases regardless the nutritional status [96]. The half-life of transferrin is approximately 8 days, higher than those of prealbumin and RBP [96]. Therefore, it is rarely used for assessing protein status.

### 3.4. Bone Status

Metabolic bone disease (MBD) typically affects infants born very prematurely. Serum calcium, phosphate, and alkaline phosphatase are commonly used to assess bone mineralization (Table 3); however, a systematic review concluded that there is insufficient evidence that these are valid early markers of MBD, using imaging methods as a reference [100].

#### 3.4.1. Serum Calcium

Serum calcium should not be used as a biochemical marker of MBD due to its poor correlation with either X-ray or DXA [100].

#### 3.4.2. Serum Phosphate

Serum phosphate <1.8 mmol/L (5.6 mg/dL) has a good correlation with DXA measurements, yielding a specificity of 96% and a sensitivity of 50% as a biochemical marker of MBD [100]. The correlation of serum phosphate <1.2 mmol/L (3.7 mg/dL) with the speed of sound measured by quantitative ultrasound is also good, yielding a specificity of 100%, sensitivity of 33%, positive predictive value of 100%, and negative predictive value of 57% for MBD [100].

#### 3.4.3. Serum Alkaline Phosphatase

As a biochemical marker of MBD, total serum alkaline phosphatase >900 U/L has a good correlation with DXA measurements, yielding a specificity of 71% and a sensitivity of 88% [100].

#### 3.4.4. Combination of Serum Phosphate and Alkaline Phosphatase

The correlation between the combination of serum alkaline phosphatase >900 U/L and phosphate <1.8 mmol/L (5.6 mg/dL) as a biochemical marker of MBD is excellent, yielding a specificity of 70% and a sensitivity of 100% [100].

#### 3.4.5. Urinary Markers

Unsatisfactory correlations have been found between calcium-creatinine ratio, urinary phosphate concentration and tubular reabsorption of phosphate as biochemical markers of MBD and radiological signs [100] or DXA measurements [101]. Formula-fed or breastfed infants have different specific patterns of urinary calcium and phosphate levels, confounding the interpretation of these markers [98].

## 4. Conclusions

The practical, valuable nutritional status assessment of preterm infants under intensive care should include valid, easily obtained, and inexpensive anthropometric measurements and clinical biochemical parameters.

Validated anthropometry parameters are useful for the postnatal diagnosis of fetal malnutrition, to monitor growth during hospital stay, and to recognize undernutrition or overnutrition early, provided accurate measurements are obtained. Measurements should be interpreted using appropriate charts: Fenton 2013 charts to assess intrauterine growth, the free-access growth calculator to monitor intra-hospital weight gain, and Intergrowth-21st standards to monitor growth after discharge up to 64 weeks postmenstrual age.

Body weight, the most frequently used isolated parameter to monitor nutritional status, does not inform on body compartments. Nevertheless, other anthropometric indices estimate the quality of growth. Body length reflects skeletal growth and is a predictor of fat-free mass; the accuracy of length measurement is of utmost importance when it is squared or cubed in indices. Head circumference indicates brain growth, but the interpretation of measurements may be affected by prematurity-related morbidity not related with nutrition. Mid-upper arm circumference is easy to measure, reproducible, and reflects quite well the variation of body adiposity. Skinfolds are convenient for bedside assessment and estimate body fat reasonably, despite not accounting for the intra-abdominal fat. Weight-to-length ratio, BMI, and ponderal index have been used to assess body proportionality at birth. These and other derived indices, such as MUAC:HC, could be convenient proxies for body composition but they are not validated.

Biochemical markers should complement the anthropometric assessment.

Low BUN correlates with insufficient protein intake, but high levels may indicate either appropriate amino acid intake, low energy intake relative to protein intake or amino acid intolerance. Prealbumin and RBP are good markers of current protein status due to their short half-lives but may be affected by factors other than protein nutrition.

The assessment of bone mineralization by serum levels of calcium, phosphate, and alkaline phosphatase is commonly used. There is insufficient evidence that, individually, these are valid biochemical markers of MBD; even though, the combination of high alkaline phosphatase and low phosphate levels is the best biochemical indicator of MBD.

## Figures and Tables

**Table 1:** Appropriate charts relating body weight with infant’s age, depending on the purpose.

Purpose	Chart/Reference Values	Characteristics
To assess intrauterine growth	Fenton 2013 [37].	Reference sex specific, cross-sectional charts. Range: 22 to 50 weeks postmenstrual age.
To monitor intra-hospital growth	Growth calculator: https://www.growthcalculator.org/ [41,42].	Reference specific for sex, gestational age and percentile, longitudinal curves.
To monitor growth after discharge	Intergrowth-21st standards [46].	Standard longitudinal curves. Range: 37 to 64 postmenstrual age.

**Table 2:** Anthropometric measurements for the assessment of nutritional status in preterm infants [12,15,16,63].

Measurement	Advantages	Limitations
Direct measurements		
Body weight	Simple and reproducible.	Does not give any information on body composition.
Body length	Reflects skeletal growth and predicts fat-free mass.	Accurate measurement is difficult.
Head circumference (HC)	Reflects brain growth.	It may be affected by causes other than nutrient intake.
Mid-upper arm circumference (MUAC)	Reflects the combined arm muscle and fat. It may estimate body adiposity.	Measurement is technically difficult in extremely preterm infants.
Skinfolds	Estimates body fat. Convenient for bedside assessment.	Do not reflect intra-abdominal fat.
Derived measurements		
Weight-to-length ratio	Reflects body proportionality at birth and postnatal body composition.	Its validity as a predictor of body composition has been questioned.
Body mass index (BMI)	Reflects body proportionality at birth and postnatal body composition. It seems more appropriate to assess body proportionality than weight-to-length ratio and ponderal index.	The reliability of BMI is highly dependent on the accuracy of length measurement. Its validity as a predictor of body composition has been questioned.
Ponderal index	Reflects body proportionality at birth and postnatal body composition.	The reliability of this index is highly dependent on the accuracy of length measurement. Its validity as a predictor of body composition has been questioned.
MUAC:HC ratio	Combined with other measurements, contributes to estimating body composition in appropriate-for-gestational age neonates.	Validation as an independent predictor of body composition is needed.
Upper-arm cross-sectional areas	They might indicate the relative contribution of fat and muscle to the total arm area better than the direct measurements.	Their ability to predict total body fat and muscle is questioned.

BMI, body mass index; HC, head circumference; MUAC, mid-upper arm circumference; MUAC:HC, mid-upper arm circumference to head circumference ratio.

**Table 3:** Biochemical markers of protein and bone status in preterm infants [8,17,18,100].

Measurement	Advantages	Limitations
Protein status		
Blood urea nitrogen (BUN)	Low BUN is a good marker of low protein intake in enterally fed, clinically stable infants.	High BUN is not easy to interpret, since it may represent appropriate amino acid intake, low energy intake relative to protein intake, or amino acid intolerance.
Serum prealbumin	Half-life of approximately 2 days. A low level reflects current protein deficit.	Inflammation or infection may decrease prealbumin levels.
Retinol-binding protein (RBP)	Half-life of approximately 12 h. A low level reflects current protein deficit.	RBP levels may be also be affected by suboptimal iron, zinc, and vitamin A status. Measuring RBP is more expensive than prealbumin, providing equivalent information.
Serum transferrin	A complementary marker of protein status.	In iron deficiency, transferrin concentration increases regardless of nutritional status. It is seldom used.
Bone status		
Serum calcium		It is a poor marker of MBD.
Serum phosphate	High specificity and positive predictive value as a marker of MBD.	Low sensitivity and negative predictive value as a marker of MBD. Insufficient evidence as a reliable marker of MBD.
Serum alkaline phosphatase	Levels >900 U/L yield a specificity of 71% and a sensitivity of 88% as a marker of MBD	Insufficient evidence as a reliable marker of MBD.
Serum alkaline phosphatase plus serum phosphate	Alkaline phosphatase >900 U/L plus phosphate <1.8 mmol/L (5.6 mg/dL) yield a specificity of 70% and a sensitivity of 100% as a marker of MBD	Insufficient evidence as a reliable marker of MBD.
Urinary calcium and phosphate markers	Urinary calcium-creatinine ratio, phosphate concentration and tubular reabsorption of phosphate may be complementarily used in the diagnosis of MBD	Levels are dependent on whether infants are formula-fed or breastfed.

BUN, blood urea nitrogen; MBD, metabolic bone disease; RBP, retinol binding protein.

## Abbreviations

%FM	percent fat mass
ADP	air displacement plethysmography
AGA	appropriate-for-gestational age
BMI	body mass index
BUN	blood urea nitrogen
DXA	dual-energy X-ray absorptiometry
HC	head circumference
LGA	large-for-gestational age
MBD	metabolic bone disease
MUAC	mid-upper arm circumference
MUAC:HC	mid-upper arm circumference to head circumference ratio
RBP	retinol binding protein
SGA	small-for-gestational age
